# Thrombocytopenia and hyperprogression after radiotherapy and camrelizumab treatment in an esophageal cancer patient with increased *JAK2* gene copies: a case report

**DOI:** 10.3389/fonc.2024.1283428

**Published:** 2024-06-21

**Authors:** Hang Wang, Yun Li, Min Qiu, Jianmin Wang

**Affiliations:** ^1^ Department of Endocrinology, Chongqing Hospital of Traditional Chinese Medicine, Chongqing, China; ^2^ College of Traditional Chinese Medicine, Chongqing Medical University, Chongqing, China; ^3^ Department of Cardiology, Hospital of Traditional Chinese Medicine in Qijiang District, Chongqing, China; ^4^ Department of Respiratory and Critical Care Medicine, The First People’s Hospital of Neijiang, Neijiang, Sichuan, China; ^5^ Department of Oncology, Chongqing Hospital of Traditional Chinese Medicine, Chongqing, China

**Keywords:** esophageal cancer, radiotherapy, immune checkpoint inhibitor, thrombocytopenia, JAK2/STAT3 pathway

## Abstract

Radiotherapy (RT) and immune checkpoint inhibitor (ICI) are important treatments for esophageal cancer. Some studies have confirmed the safety and effectiveness of using RT in combination with ICI, while serious side effects have been exhibited by some patients. We report a patient with metastatic esophageal cancer who received RT combined with ICI. The patient experienced severe thrombocytopenia, and treatment with thrombopoietin and corticosteroids were ineffective. Finally, the patient developed abscopal hyperprogression outside the radiation field. Interestingly, next-generation sequencing revealed increased JAK2 gene copies in the surgical slices. The JAK2/STAT3 pathway is involved in the regulation of megakaryocyte development. Recurrent thrombocytopenia may activate the JAK2/STAT3 pathway, leading to megakaryocyte differentiation and platelet biogenesis. However, persistent activation of the JAK2/STAT3 pathway has been associated with immune ICI resistance and tumor progression. This case indicates that thrombocytopenia and increased JAK2 gene copies may be risk factors for poor prognosis after ICI and RT treatment.

## Introduction

According to GLOBOCAN 2022 estimates, esophageal cancer was the eleventh most commonly diagnosed cancer and the seventh leading cause of cancer-related death worldwide in 2022 (1). Different standard therapies are recommended according to clinical stage and pathological pattern. In early-stage esophageal cancer, surgery remains the mainstay treatment, whereas for locally advanced esophageal cancer, chemoradiotherapy is the standard approach. In addition to chemotherapy—the current standard first-line treatment—several FDA-approved targeted therapeutic agents are available for advanced or metastatic esophageal cancer.

In the wake of inspiring data from many clinical studies, immune checkpoint inhibitors (ICIs) are now recommended as first- and second-line therapies for advanced esophageal cancer. The KEYNOTE181 (2), ATTRACTION-3 (3), and ESCORT (4) studies confirmed that the overall survival (OS) of advanced esophageal cancer patients was prolonged with ICI therapy versus chemotherapy. Therefore, pembrolizumab, nivolumab, or camrelizumab combined with chemotherapy have been proposed as first-line therapies or alone as second-line therapies for advanced esophageal cancer patients according to the 2021 National Comprehensive Cancer Network (NCCN) and the Chinese Society of Clinical Oncology (CSCO) guidelines. Although ICI therapy is associated with fewer side effects than chemotherapy, immune-related adverse events (irAEs) are not unusual and include pneumonia, hepatitis, nephritis, endocrinopathy, and hematotoxicity, among others (5).

Radiotherapy (RT) is an important treatment for resectable and unresectable esophageal cancer (6). The combination of RT and ICI therapy may have a synergistic effect on the antitumor immune response (7). RT induces apoptosis of tumor cells, thereby increasing tumor antigen release and cross-presentation via antigen-presenting cells (APCs). Specifically, RT enhances the inflammatory immune response and subsequently improves the immunosuppressive tumor microenvironment, which ultimately potentiates the efficacy of ICI. Some studies have confirmed the safety and effectiveness of RT plus ICI. A meta-analysis including 11 studies (8) indicated that patients treated with ICI combined with RT have better objective response rates (ORRs) and disease control rates (DCRs) than those treated with single ICI (42% *vs*. 15% and 85% *vs*. 26%). Another meta-analysis including 51 studies (9) revealed comparable grade 3–4 toxicity between ICI plus RT and ICI alone (16.3% *vs*. 22.3%, for ICI plus RT and ICI alone, respectively). Even so, some patients still exhibit severe side effects or progressive disease (PD) after ICI therapy plus RT. In the current study, we report an unusual case in which radioimmunotherapy resulted in severe thrombocytopenia and hyperprogression.

## Case presentation

**Figure 1:**
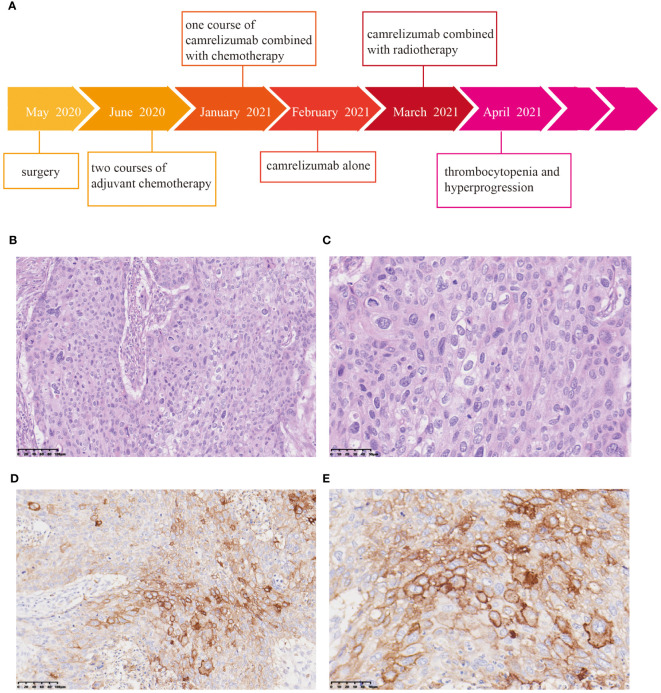
Pathological and immunohistochemical examination of surgical specimens. **(A)** Timeline of the treatment process. Hematoxylin and eosin (HE) staining revealed moderate to poorly differentiated squamous cell carcinoma. Magnification: 20× **(B)** and 40× **(C)**. Immunohistochemistry showed positivity for PD-L1. Magnification: 20× **(D)** and 40× **(E)**.

In May 2020, a 62-year-old man presented with a 2-month history of dysphagia, and mucosal biopsy performed via gastroscopy revealed thoracic esophageal squamous cell carcinoma (ESCC). The main course of diagnosis and treatment of the patient is shown in **Figure 1A**. Radical esophagectomy was subsequently performed. Pathological examination confirmed moderate to poorly differentiated esophageal squamous cell carcinoma, and the pathological stage was determined to be T2N0M0 IB (**Figures 1B, C**). Two courses of adjuvant chemotherapy consisting of docetaxel (75 mg/m^2^) and lobaplatin (75 mg/m^2^) were administered every 3 weeks. Then, stage 3 myelosuppression (mainly thrombocytopenia) occurred, at which point the administration of recombinant human thrombopoietin (TPO) rapidly improved the platelet count. The patient then received a modified third round of chemotherapy consisting of docetaxel (75 mg/m^2^) and cisplatin (75 mg/m^2^). However, the patient experienced severe thrombocytopenia, and chemotherapy was discontinued. In January 2021, the patient again experienced mild dysphagia, and a CT scan revealed metastases in the liver and cervical lymph node as well as gastroesophageal anastomosis. Since positive PD-L1 expression was observed in the tumor tissue [10% of the tumor cell proportion score (TPS) and 20% of the combined positive score (CPS)] (**Figures 1D, E**), ICI was considered. Camrelizumab combined with cisplatin (75 mg/m^2^) and 5-fluorouracil (400 mg/m^2^) was administered on January 31, 2021. Unfortunately, the patient again experienced grade 4 thrombocytopenia, which was cured upon TPO administration. However, chemotherapy had to be completely suspended because of recurrent hematological toxicity. On February 14, 2021, the patient began receiving treatment consisting of 200 mg camrelizumab alone every 2 weeks. The patient’s platelet count was normal during this period.

**Figure 2:**
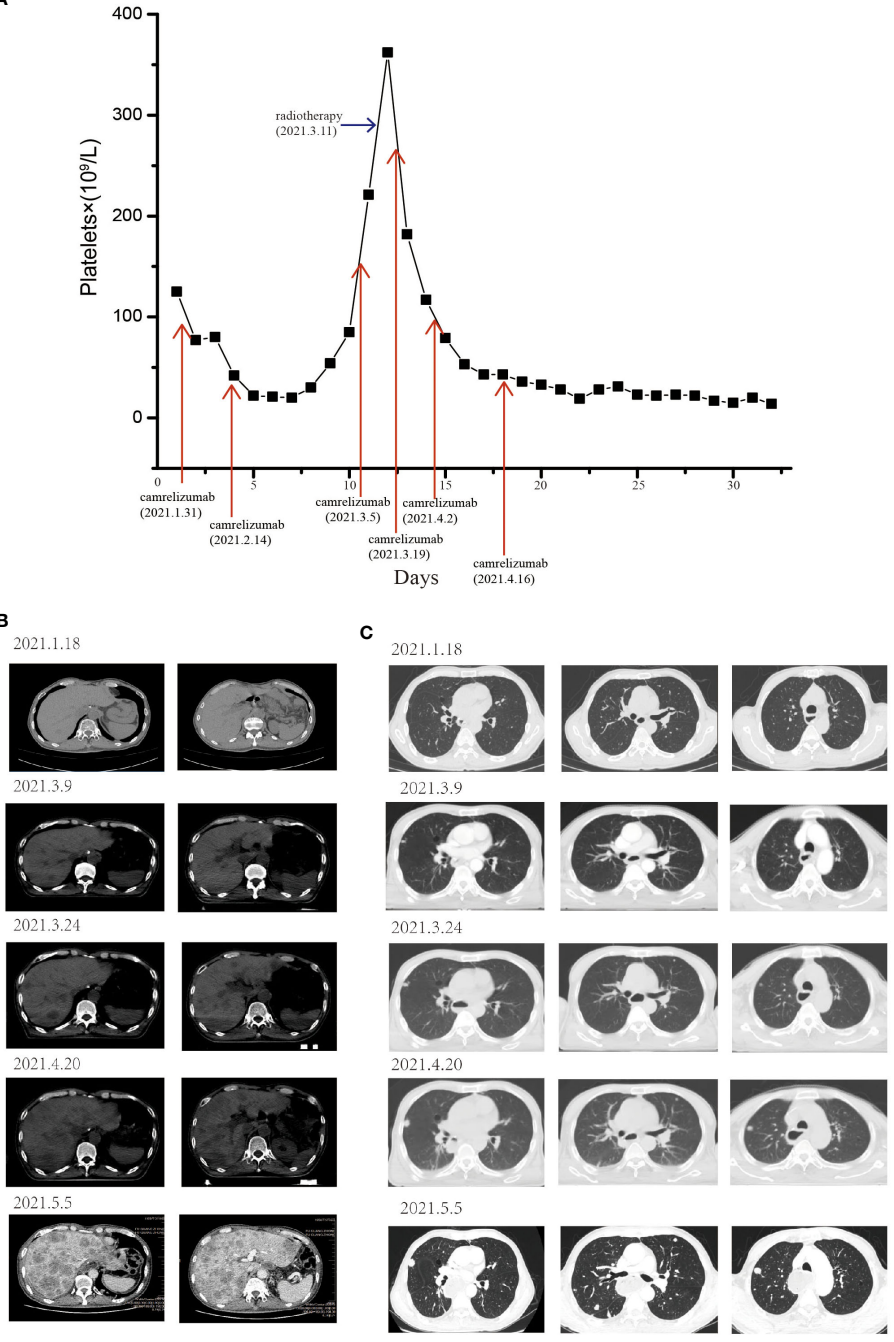
The patient experienced severe thrombocytopenia and hyperprogression after RT combined with camrelizumab treatment. **(A)** Longitudinal changes in platelet count over time. The pulmonary and liver metastases before and after RT combined with ICI. After receiving RT and camrelizumab on March 11, 2021, the development of liver **(B)** and pulmonary **(C)** metastases rapidly increased, and new metastases appeared.

Furthermore, the patient experienced progressive dysphagia, and symptom relief was our responsibility. Palliative management of dysphagia can be achieved through multiple modalities. Esophageal stent placement (10) and radiotherapy (11, 12) could be used for palliation of dysphagia symptoms. After communication with the patient, intensity-modulated radiotherapy (54 Gy in 28 fractions) was administered to the cervical lymph nodes and gastroesophageal anastomosis starting on March 11, 2021. After treatment with ICI combined with RT, the platelet count rapidly decreased (<25×10^9^/L) (**Figure 2A**), and TPO was ineffective at treating this condition. Bone marrow aspiration excluded a central etiology. Camrelizumab-induced immune thrombocytopenia could not be excluded, and oral corticosteroids (1 mg/kg/day) were then started in combination with TPO when the platelet count reached 20×10^9^/L. However, the patient’s platelet count did not recover.

**Figure 3:**
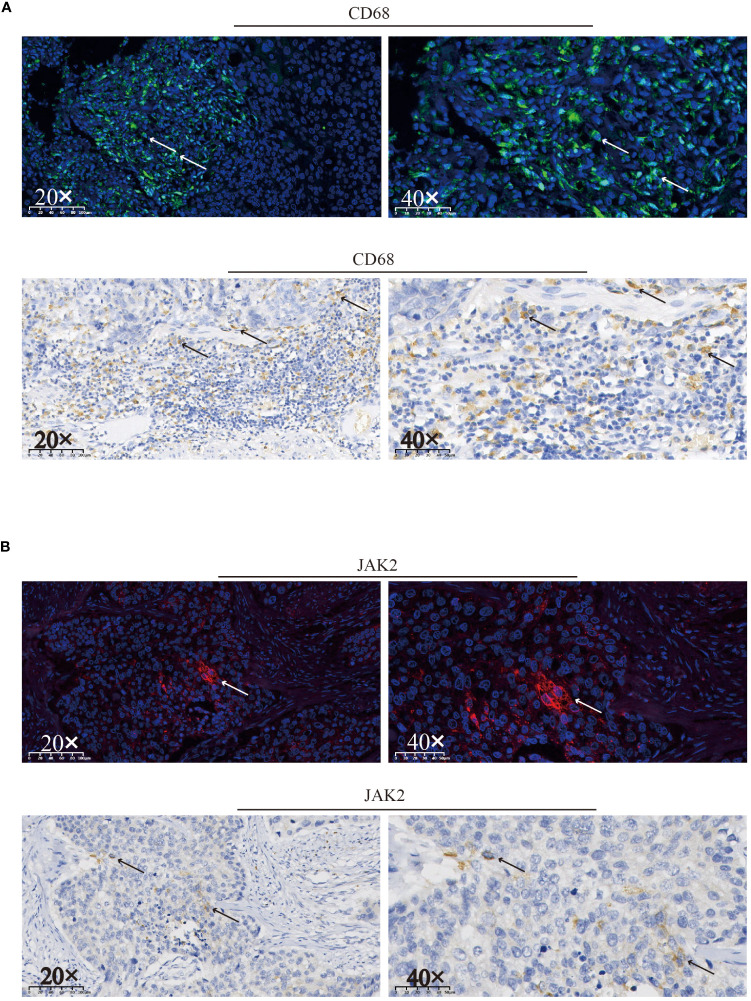
Immunofluorescence and immunohistochemical staining were used to assess the expression of CD68 **(A)** and JAK2 **(B)** and in surgical specimens. Positive staining for CD68 or JAK2 is indicated by arrows on the respective slides.

The patient experienced chemotherapy-induced thrombocytopenia due to the direct destructive effects of cytotoxic agents, and treatment with TPO facilitated rapid recovery. However, after RT and ICI treatment, the platelet count continuously decreased, and no response to TPO or corticosteroids was observed. Macrophage-mediated platelet destruction resulting from abnormal autoantibody activation may also occur (13). An abundance of CD68^+^ macrophages were detected in surgical specimens by immunohistochemistry (IHC) and immunofluorescence (IF) (**Figure 3A**). Furthermore, to analyze the potential cause and prognosis, we performed next-generation sequencing (NGS) of DNA from surgical specimens. Mutations in the *TP53* gene at position p.L114A, along with increases in copy number for both the *KRAS* and *JAK2* genes were identified (**Table 1**). We detected and confirmed the positive expression of JAK2 by IHC and IF in tumor cells from surgical specimens (**Figure 3B**).

**Table 1:** Next-generation sequencing analysis of DNA from surgical specimens.

Gene	Detection result	Mutation abundance/copies
*TP53* *JAK2* *KRAS*	p.L114Afs*34 Exon4 increased copies increased copies	57.93% >20 >20

The patient’s dysphagia was alleviated by RT, but a CT scan revealed some new lesions in the lung and metastases in the liver, which progressed gradually. Since the end of April 2021, the patient had become increasingly tired and exhibited polypnea and rapid disease progression. On May 5, 2021, a CT scan revealed diffuse liver lesions and multiple metastases in the lung (**Figures 2B, C**). Finally, the patient developed multiple organ failure and died.

## Discussion

It is well known that RT interacts with the host immune system. RT causes the release of tumor antigens, which leads to increased tumor antigen presentation, thereby recruiting and activating antitumor subsets, such as CD4^+^ and CD8^+^ T cells, cytotoxic NK cells, and CD8^+^ CD56^+^ natural killer T (NKT) cells (14). Thus, the low-immunogenicity tumor microenvironment converts to an immune-active state. On the contrary, RT can deplete cytotoxic T cells by inducing exhaustion signals, such as PD-1 and Tim-3, and by increasing TGF-β, which suppresses T cell activation and fosters regulatory T cell development. Hence, tumor cells can subvert immunosurveillance, which eventually results in radioresistance (15). Ioannis M Koukourakis (16) reported that a bladder cancer patient developed abscopal hyperprogression outside the radiation field after receiving RT combined with anti-PD-1 immunotherapy. In the present study, concurrent RT and biweekly anti-PD-1 therapy resulted in abscopal hyperprogression as well as severe hematological side effects.

Hyperprogression is observed in some patients who receive ICI treatment. Some researchers have defined hyperprogression as 1) treatment failure within 2 months, 2) an increase in target lesions >50%, 3) substantial clinical deterioration or 4) the appearance of 2 or more new lesions or new organ involvement compared with the most recent scan (17). The physiopathological mechanisms of hyperprogression that occurs during ICI therapy include primary resistance to immunotherapy, lack of targets, exhaustion of T cells, modulation of tumor-promoting cells, aberrant inflammation, and activation of oncogenic pathways (18). A retrospective study indicated a significant decrease in circulating albumin and increases in white blood cell count and c-reactive protein after nivolumab treatment in the hyperprogression group compared with the progression group in patients with non-small lung cancer (19). However, more rigorous studies as well as more observations and discussions are needed to demonstrate whether these biomarkers can predict hyperprogression. The patient experienced rapid disease deterioration after administration of camrelizumab combined with RT. The time-to-treatment failure (TTF) was less than 2 months, the lesion outside the RT field increased rapidly, and new metastases were observed. Thus, we classified the progression as abscopal hyperprogression following the combination treatment of RT and ICI.

Moreover, the patient experienced severe thrombocytopenia within a short time after receiving RT plus ICI. ICI-induced immune thrombocytopenia can be a serious and life-threatening adverse event that is sometimes associated with poor clinical outcomes (20, 21). Patients with grade 3 or higher ICI-related thrombocytopenia experience worse OS than patients without thrombocytopenia of any etiology (4.17 *vs*. 13.31 months) (21). Treatment options include corticosteroids, intravenous immune globulin, a thrombopoietin receptor agonist, and rituximab. The pathophysiologic mechanism of ICI-induced thrombocytopenia is also unclear. Reinvigoration of CD4^+^ and CD8^+^ T cells after ICI treatment, along with activation of inflammatory pathways, may lead to autoimmune destruction of hematopoietic stem cells (20). In the patient described here, TPO rapidly improved the thrombocytopenia associated with chemotherapy but was ineffective in ameliorating the patient’s condition following RT combined with camrelizumab. We found previous reports of fatal immune thrombocytopenia related to ICI administered around the time of RT. Notably, the reported patients displayed no hematological irAEs during treatment with mono-ICI, yet a rapid decrease in platelet count was observed following intracranial radiation (22, 23). RT induces tumor cell apoptosis and inflammation, which may activate macrophages that can mediate platelet destruction. PD-1 antibodies can bind to PD-1 and block the PD-L1/PD-1 and PD-L2/PD-1 signaling pathways, the latter of which is correlated with immune tolerance. Once immune tolerance is disrupted, the mononuclear phagocyte system is activated, which leads to platelet destruction. Prominent infiltration of PD-L1-positive M2 macrophages was detected in a metastatic lymph node in a gastric cancer patient with hyperprogression after nivolumab therapy (24). We detected macrophage infiltration in surgical specimens, but since we did not obtain tumor tissues after RT plus camrelizumab treatment, we could not evaluate changes in macrophage infiltration post treatment.

Furthermore, NGS confirmed elevated *JAK2* copy number, and expression of the JAK2 protein in surgical specimens was shown by IHC and IF. The *JAK2* gene encodes a tyrosine kinase that plays a pivotal role in JAK2/STAT3 signaling, which is predominantly involved in the regulation of megakaryocyte development, thereby promoting thrombocytopoiesis (25). In addition to the intrinsically increased number of *JAK2* copies, recurrent thrombocytopenia and TPO application might activate the JAK2/STAT3 signaling pathway as a feedback mechanism to stimulate thrombopoiesis. On the contrary, *JAK2* gene amplification may cause persistent activation of the JAK2/STAT3 pathway, which has been linked to survival, proliferation, angiogenesis, resistance to apoptosis, carcinogenesis, and metastasis in many types of human cancer cells (26). Moreover, activation of the JAK2/STAT3 pathway causes PD-L1-mediated immune escape by reducing T-cell activation, metabolic activity, and cell cycle progression, which results in resistance to ICI (27). That is, recurrent thrombocytopenia and increased *JAK*2 copy number are indicative of a poor prognosis. For the same reason discussed above, we were unable to detect alterations in the JAK2/STAT3 signaling pathway after ICI- and RT-induced thrombocytopenia.

In addition, the patient harbored a *TP53* mutation abundance of 57.93%. Loss of the powerful tumor suppressor *TP53* might be correlated with poor overall survival in patients with esophageal cancer (28). Indeed, *TP53* has regulatory interactions with other tumor-related genes. Immunohistochemical analysis of human colorectal cancer samples revealed a correlation between missense mutations in *TP53* (mutp53) and high levels of activated pJAK2 and pSTAT3 (29). In pancreatic tumors, loss of p53 function activated the JAK2-STAT3 signaling pathway (30). In conclusion, stabilized mutp53 expression activates JAK2/STAT3 signaling, drives cell cycle progression and induces proliferation and invasion.

Here, we report a patient with esophageal squamous cell carcinoma who developed severe thrombocytopenia immediately following RT and camrelizumab treatment and who experienced rapid abscopal hyperprogression. ICI may provoke an aberrant autoimmune response that is exacerbated by radiotherapy, leading to a condition that facilitates macrophage activation, and consequently, thrombocytopenia. Moreover, elevated *JAK2* copy number and aberrant activation of the JAK2/STAT3 pathway, which are negative feedback pathways for thrombocytopenia, contribute to platelet biogenesis and immune escape. *TP53* mutations may activate the JAK2/STAT3 pathway, which would result in tumor hyperprogression. We cannot confirm the direct correlation between thrombocytopenia and hyperprogression. Prospective studies are needed to demonstrate any modifications of the JAK2/STAT3 pathway following thrombocytopenia and to determine whether the JAK2/STAT3 pathway may contribute to hyperprogression. Survival analyses between patients with activated JAK2/STAT3 plus mutated *TP53* and those with activated JAK2/STAT3 alone should be performed in future studies.

## Data availability statement

The original contributions presented in the study are included in the article/supplementary material. Further inquiries can be directed to the corresponding authors.

## Ethics statement

The studies involving humans were approved by Ethics Committee of Chongqing Hospital of Traditional Chinese medicine. The studies were conducted in accordance with the local legislation and institutional requirements. The human samples used in this study were acquired from the surgical sections of necessary esophagectomy. Written informed consent for participation was obtained from the participants’ wife in accordance with the national legislation and institutional requirements. Written informed consent was not obtained from the individual(s) for the publication of any potentially identifiable images or data included in this article because the patient was dead before we written the manuscript, but we acquired written informed consent of his wife.

## Author contributions

HW: Writing – original draft. YL: Writing – original draft. MQ: Supervision, Writing – review & editing. JW: Funding acquisition, Supervision, Writing – review & editing.
